# A draft conceptual model of SLC6A1 neurodevelopmental disorder

**DOI:** 10.3389/fnins.2022.1026065

**Published:** 2023-01-19

**Authors:** Kimberly Goodspeed, Lindsay R. Mosca, Nicole C. Weitzel, Kyle Horning, Elijah W. Simon, Anna C. Pfalzer, Maya Xia, Katherine Langer, Amber Freed, Megan Bone, Maria Picone, Terry Jo V. Bichell

**Affiliations:** ^1^Department of Pediatrics, University of Texas Southwestern Medical Center, Dallas, TX, United States; ^2^College of Arts and Sciences, Vanderbilt University, Nashville, TN, United States; ^3^COMBINEDBrain, Brentwood, TN, United States; ^4^SLC6A1 Connect, Denver, CO, United States; ^5^TREND Community, Philadelphia, PA, United States

**Keywords:** SLC6A1, GAT1, epilepsy, rare disease, natural history, disease concept, social listening, outcome measures

## Abstract

**Introduction:**

*SLC6A1* Neurodevelopmental Disorder (SLC6A1-NDD), first described in 2015, is a rare syndrome caused by a mutation in the *SLC6A1* gene which encodes for the GABA Transporter 1 (GAT-1) protein. Epilepsy is one of the most common symptoms in patients and is often the primary treatment target, though the severity of epilepsy is variable. The impact of seizures and other symptoms of SLC6A1-NDD on patients and caregivers is wide-ranging and has not been described in a formal disease concept study.

**Methods:**

A literature search was performed using the simple search term, “SLC6A1.” Papers published before 2015, and those which did not describe the human neurodevelopmental disorder were removed from analysis. Open-ended interviews on lived experiences were conducted with two patient advocate key opinion leaders. An analysis of de-identified conversations between families of people with SLC6A1-NDD on social media was performed to quantify topics of concern.

**Results:**

Published literature described symptoms in all of the following domains: neurological, visual, motor, cognitive, communication, behavior, gastrointestinal, sleep, musculo-skeletal, and emotional in addition to epilepsy. Key opinion leaders noted two unpublished features: altered hand use in infants, and developmental regression with onset of epilepsy. Analysis of social media interactions confirmed that the core symptoms of epilepsy and autistic traits were prominent concerns, but also demonstrated that other symptoms have a large impact on family life.

**Discussion:**

For rare diseases, analysis of published literature is important, but may not be as comprehensive as that which can be gleaned from spontaneous interactions between families and through qualitative interviews. This report reflects our current understanding of the lived experience of SLC6A1-NDD. The discrepancy between the domains of disease reported in the literature and those discussed in patient conversations suggests that a formal qualitative interview-based disease concept study of SLC6A1-NDD is warranted.

## Introduction

The solute carrier family 6 member 1 (*SLC6A1*) neurodevelopmental disorders (SLC6A1-NDD) are a rising cause of epilepsy, developmental disability, and autism spectrum disorder (ASD) (Goodspeed et al., 2020). The *SLC6A1* gene was first identified in a cohort of seven patients with epilepsy with myoclonic atonic seizures (EMAS) in 2015 (Carvill et al., 2015). However, in the 5 years following the initial publication, another 327 cases were identified, and the prevalence of SLC6A1-NDD is now estimated to be fairly high, with current calculations placing the incidence at 1 in 38,000 births (López-Rivera et al., 2020). As additional cases have been described, the spectrum of disease and symptom severity has continued to evolve and expand. It is a crucial time point to develop a conceptual model of disease for SLC6A1-NDD. A conceptual model, or disease concept map, is a comprehensive description of the lived experience of people with a disorder derived from comprehensive open-ended qualitative interviews of patients, caregivers and health care providers (Grieco et al., 2019; Willgoss et al., 2020; Staunton et al., 2022; Sullivan et al., 2022). A disease concept map can serve as the basis for standards of clinical care, natural history study design, and endpoints for clinical trials (Gonzalez-McQuire et al., 2019). The first stage in the process is a “draft conceptual model,” which is built on published literature and interviews with a few patient advocates. The draft is then expanded, refined, or changed after the qualitative study is complete. The draft model of SLC6A1-NDD reported here also takes into account a novel analysis of social media, which may make it more robust than a model based substantially on literature.

The *SLC6A1* gene encodes the voltage dependent gamma-aminobutyric acid (GABA) transporter (GAT-1) which is responsible for GABA reuptake from the synaptic cleft and is predominantly expressed in the nerve terminal of GABAergic interneurons in the brain as well as astrocytes (Madsen et al., 2015). There are more than 100 known *SLC6A1* variants (Cai et al., 2019), and the vast majority of these occur *de novo* and affect highly conserved nucleotide sequences (Madsen et al., 2015). SLC6A1-NDD occurs mainly from missense mutations in the *SLC6A1* gene, which result in a loss of function for GAT1 (Cai et al., 2019). An analysis of patients with SLC6A1-NDD reveals that many of these variants affect amino acid residues located within the transmembrane domain of the GAT-1 protein (Palmer et al., 2016). These variants impair GAT-1 localization to the cell membrane (Cai et al., 2019). Additionally, variants which change the hydrophobicity of amino acid residues (i.e., substitution of a hydrophobic for a hydrophilic residue) likely de-stabilize the GAT-1 protein structure, resulting in mis-folding and aggregation in the endoplasmic reticulum (Wang et al., 2020). These impairments in protein folding and trafficking ultimately lead to diminished clearance of GABA from the synapse (Wang et al., 2020). Mutations related to GAT1 gain of function are likely to be related to transporter function rather than localization (Ahring et al., 2022). It remains unclear how dysregulation of synaptic GABA levels in the context of mutations in *SLC6A1* result in the observed clinical phenotypes. Furthermore, there is no clear genotype-phenotype correlation between mutational locations and clinical symptom or symptom severity in SLC6A1-NDD (Johannesen et al., 2018).

As novel therapeutic interventions are being developed, a deeper understanding of the impact of the full spectrum of SLC6A1-NDD severity on patients and caregivers is needed. To aid in the design of natural history studies and identification of clinical trial endpoints, a draft disease concept map of SLC6A1-NDD was created based on a survey of the literature, an analysis of social media interactions, and interviews with two key opinion leaders (KOLs). Due to the relatively new discovery of SLC6A1-NDD, no formal conceptual model has yet been published. SLC6A1 is included in the list of genes for the Simons Searchlight patient registry which is a large, longitudinal study of genetic causes of autism spectrum disorder through remote collection of medical and genetic information, and standardized questionnaires completed by caregivers (Kahen et al., 2021).

Though there is a paucity of published literature on the clinical description of SLC6A1-NDD, on social media there is an interactive group of families of SLC6A1-NDD patients in a private Facebook group. New approaches can analyze conversations on social media platforms to gain insights into individuals’ reported life experiences (Patel et al., 2019; Picone et al., 2020). In this case, social media review was used to gain insight into the caregiver experience. TREND Community is a biotech company that aims to leverage real world data to aid in discovery and innovation of novel treatment for rare and chronic disorders. TREND Community conducted an analysis of the SLC6A1 Facebook group, which consisted of 265 members. Conversations between these families are an uncensored source of information, and posted comments can be analyzed quantitatively for domains and themes that are repeatedly discussed. This analysis, performed by TREND Community, reveals the areas of highest concern among parents and caregivers of people with SLC6A1-NDD, and is additive to the information gleaned from a literature search.

Following guidance from the U. S. Food and Drug Administration, a conceptual model of SLC6A1-NDD must include information obtained from open-ended qualitative interviews of patients, caregivers, and healthcare providers (U.S. Department of Health and Human Services FDA Center for Drug Evaluation and Research et al., 2006), with domains of disease described in terms of symptoms as well as the impact of these symptoms on patients and caregivers (Willgoss et al., 2020). Conceptual models serve to aid in the design of natural history studies and in the preparation of clinical trial endpoints. The draft conceptual model reported here, while not drawn from a comprehensive set of qualitative interviews, reflects current understanding of the neurodevelopmental disorders and developmental epileptic encephalopathies (DEE) caused by mutations in *SLC6A1*, based upon all cases reported in the published literature, supplemented by an analysis of interactions between caregivers on social media, and interviews with two KOLs. This draft conceptual model of SLC6A1-NDD will serve as a foundation on which to build a comprehensive conceptual model in the future, with the addition of qualitative interviews of patients, caregivers, and health care providers.

## Materials and methods

### Literature search

A literature search on PubMed was performed on July 10, 2022, using the simple search term “SLC6A1,” resulting in 436 papers, of which the earliest was published in 1990. Those papers which did not describe human patients were set aside, leaving 65 papers which discussed human patients and their symptoms. Among these 65 papers, 18 had been published before the neurodevelopmental disorder was identified, thus were focused on other phenotypes. The remaining 47 papers were published after the seminal paper in 2015 which described seven epileptic patients with *SLC6A1* variants (Carvill et al., 2015). Of the 47 clinically relevant papers, two were focused on attention deficit disorder (Merriman et al., 2015; Yuan et al., 2017, p1), two researched a connection with alcoholism (Adkins et al., 2015; Enoch et al., 2016), one found *SLC6A1* variants related to Alzheimer’s disease (Zhu et al., 2020), one focused on connections to anxiety (Sarris et al., 2020), three were focused on somatic mutations in malignant tumors (Eshragh et al., 2017; Chen et al., 2020; Wang et al., 2022, p1), one focused on dystonia (Zech et al., 2017), one included multiple gene deletions (Dikow et al., 2014; Yuan et al., 2020), four reported *SLC6A1* variants associated with schizophrenia (Frankle et al., 2015; Hoftman et al., 2015; Zhao et al., 2016; Rees et al., 2020), three focused on other similar mental health disorders (Demarest et al., 2019; Ahring et al., 2022; Knight et al., 2022), two described genetic testing technologies (Schijns et al., 2020; Salinas et al., 2021), and two described general developmental and epileptic encephalopathies (DEEs) without specific description of patients with *SLC6A1* mutation (Schousboe et al., 2004; Guillen-Guio et al., 2018; Galer et al., 2020; Salinas et al., 2021). Only 25 papers included clinical description of patients with neurodevelopmental symptoms and DEEs, with a likely overlap of a few patients between these papers (Supplementary Table 1).

We compiled a listing of patients described in each of these papers (Supplementary Table 2), with the symptoms categorized for each patient according to a template derived from Willgoss et al. (2020). The list of patients was derived from the 25 clinically relevant publications, eliminating patients who had been previously described in other publications. It is possible that some of reports of patients were duplicates of patients previously mentioned. Column E of the Supplementary Table 1 describes the reason for including or excluding a patient from the list, while Column G enumerates the estimated unique cases and Column H lists the total number of cases discussed in each paper. Relative frequency of these symptoms was calculated for those symptoms mentioned in the literature, and again after addition of those gleaned from social media review.

### Social media data

TREND Community obtained permission to analyze anonymized conversations from a private Facebook group for caregivers of individuals with SLC6A1-NDD between Jan 2016 and Feb 2021. The Facebook group “SLC6A1 Family Support Group” defines itself as a group for support and encouragement. All conversations were de-identified to ensure anonymity. The Western Institutional Review Board issued an exemption under 45 CFR §46.104(d) (4) for the social media conversation analysis (listening). De-identified Facebook conversations were analyzed separately by a proprietary analytics engine, “Krystie” (TREND Community, Philadelphia, PA, United States), to quantify the number of mentions of specific terms or phrases as previously described (Pullen et al., 2019). “Krystie” is a natural language processing system that specializes in recognizing terms and phrases as they relate to health from social media content.

### Interviews

Key opinion leaders, who are parents of children with SLC6A1-NDD, were interviewed using open-ended questions. Neither KOL was a health professional. Both had previously reviewed the literature-based conceptual model and were knowledgeable about the purpose of the model, hence the impacts they described may be eliminated or enhanced in the final model. Additional symptoms and impacts that were described by these KOLs were added to the conceptual model. Both KOLs had previously consented to be contacted by COMBINEDBrain staff about SLC6A1-NDD through IRB# NB200058 approved August 1, 2022 by NorthStar IRB. During the interviews, the interviewers took notes about symptoms and domains mentioned, but the interviews were not recorded.

## Results

A draft disease concept map for SLC6A1-NDD was prepared in a format derived from Willgoss et al. (2020) based on domains of disease identified through published literature (Table 1). The results of the Community Voice Report were incorporated into this model. Subsequently, interviews of two KOLs were conducted, and the additional domains and impacts they described were added to this draft conceptual model.

**TABLE 1:** Draft disease concept map for SLC6A1 neurodevelopmental disorder (SLC6A1-NDD).

Neurological	Motor	Cognitive	Communication	Behavioral	Gastrointestinal
Ataxia	Fatigue	Attention deficit	Expressive communication decreased	Autism/ Autistic traits	Constipation
Electroencephalogram (EEG) abnormal	Fine motor delayed/ impaired	Cognitive decline/ regression (loss of skills)	Receptive communication decreased	Easily excitable, hyperactive, impulsive, restless	Diarrhea
Hypotonia	Gross motor delayed, impaired	Cognitive impairment (stable disability)	Non-Verbal communication	Food avoiding/ seeking behaviors	Drooling
Neuropathies (mild)	Gait abnormality	Judgment impaired	Regression in communication skills	Laughter inappropriate	Gagging
Seizures (absence, atonic, and myoclonic)	Altered hand use, and visual fixation on their fingers	Memory challenges	Emotional	Maladaptive behaviors	Incontinence
Tremor	Oral motor Impairment	Musculo-skeletal	Aggression	Obsession/ fascination with sensory items	Reflux
Visual	Regression in motor skills	Craniofacial abnormalities	Anxiety	Pica (eating non-food items)	Other
Nystagmus	Sleep	Dental issues	Frustration	Repetitive behaviors	Ear infections (childhood)
Visual-spatial issues (includes depth perception)	Disturbance of sleep in general	Scoliosis	Panic attacks	Social inappropriate/ lacking social inhibition	Hypopigmented
				Temper tantrums	Sensitivity to heat

Domains of disease describing the symptoms of SLC6A1 Neurodevelopmental Disorder (SLC6A1-NDD), based on published literature, issues identified through the TREND Community Voice Report (CVR), and concepts elicited in open-ended interviews with two key opinion leaders (KOL). Domains of disease for people with SLC6A1 mutation include Neurological, Motor, Cognitive, Communication, Behavioral, Gastrointestinal, Visual, Sleep, Musculoskeletal, and Other. Each domain includes specific symptoms reported. These domains of disease are not listed by frequency nor ranked for severity. The Gastrointestinal and Musculoskeletal domains and much of the Behavior domain were reported by the CVR and the KOL interviews but not in the literature. (Gray scale is used for visual purposes only, to delineate separate categories).

The draft conceptual model illustrates that patients with SLC6A1-NDD manifest a broad range of symptoms which impact many aspects of physical and mental health. There are 11 primary symptom domains which result in many patient and caregiver impacts (Tables 2, 3) including Neurological, Motor, Cognitive, Communication, Behavioral, Gastrointestinal, Visual, Sleep, Musculoskeletal, and Other.

**TABLE 2:** Individual impacts of SLC6A1-related neurodevelopmental disorder.

Proximal impacts	Distal impacts	
**Self-care and daily living skills**	**Community/School**	**Socialization**
Activities of daily living (lacks independence)	Absenteeism	Behaviors challenging in social situations
Bathing assistance needed (includes all hygiene)	Academic challenges (requires educational supports)	Impulsive or inappropriate social interactions
Dressing assistance needed	Communication (difficulty being understood outside family)	Social isolation
Eating assistance needed	Safety (decreased recognition of danger)	Sports/playing with others is difficult or impossible
Falls and injuries	Transitioning issues (difficulty adapting to change)	Work (difficult or impossible to work productively)
Household tasks assistance needed		
Toileting assistance needed		

**TABLE 3:** Caregiver impacts of SLC6A1-related neurodevelopmental disorder.

Caregiver and family burden		Modifying factors
Anxiety and stress	Injuries (risks higher due to assisting child with seizures, behavior, and impulse control)	Access to therapy and adaptive equipment
Depression and guilt	Intimacy impacted	Coping strategies
Destruction of possessions and property by child	Medical and therapy appointments increased due to issues caused by child’s disorder	Support services, specifically child-care
Fatigue, exhaustion, and disrupted sleep	Mobility reduced due to caregiving responsibilities	
Financial burden	Siblings and family interactions impacted	
Formal support system needed	Socialization and leisure activities reduced	
Household tasks difficult to complete	Work, reduced capacity	

As expected, under the neurological domain, seizures are a common feature of SLC6A1-NDD. In a 2018 study focused on the phenotypic spectrum of *SLC6A1* variants, 91% of patients experienced epilepsy, representing a hallmark symptom of this disorder (Johannesen et al., 2018). The most prevalent seizure semiologies included absence seizures, followed by atonic and myoclonic seizures, and seizures typically develop between 8 months and 6 years old (Goodspeed et al., 2020). Individuals with SLC6A1-NDD exhibit other neurological symptoms, including mild motor neuropathies observed using electromyogram-nerve conduction studies (EMG-NCS), tremors, hypotonia, and ataxia. Other symptoms include problems related to cognition, communication, motor function, sleep, behavioral difficulties, and gastrointestinal issues. Clinicians and caregivers also report notable reductions in both expressive and receptive communication skills, with adaptive use of non-verbal communication through facial expressions and body gestures.

Symptoms of SLC6A1-NDD typically present in childhood, first coming to medical attention with developmental delay (Carvill et al., 2015). The rate of developmental delay is not yet well defined, but many children meet their early motor and language milestones later than typically developing children. Cognitive disability was noted in 97% of patients, with over 80% of these patients categorized with mild to-moderate intellectual disability (Johannesen et al., 2018). Increased severity of developmental disability was noted after seizure-onset in a retrospective review of individuals with SLC6A1-NDD (Goodspeed et al., 2020). Cognitive impairment denotes permanent and stable issues with cognition, and is distinguished from cognitive decline/regression, in which skills and abilities are lost over time. Both concepts are reported to be features of SLC6A1-NDD. SLC6A1-NDD is also associated with a broad spectrum of maladaptive behaviors ranging from food-related and eating behaviors, hyperexcitability and inappropriate laughter, to sensory problems and impulsivity. Autism and autistic features such as stereotypy and impaired social interactions are another common symptom of the disorder (Goodspeed et al., 2020; Kahen et al., 2021; Ahring et al., 2022; Bain et al., 2022; Fischer et al., 2022). One of the first signs of the disorder, which is not in the literature, but was reported by both KOLs, is a child’s visual fixation on their own hands, and an inability to use their hands in a productive capacity, listed as “altered hand use” in the conceptual model.

**FIGURE 1:**
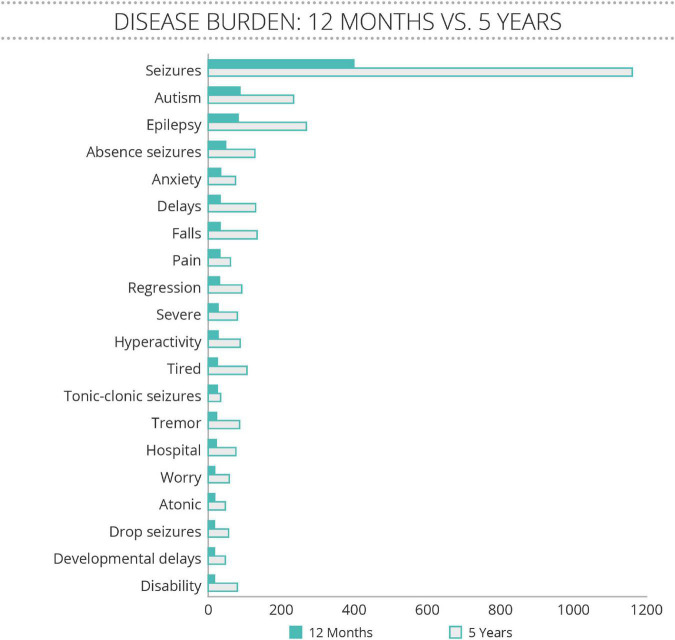
Disease Concepts most often mentioned spontaneously by family members of people with SLC6A1 Neurodevelopmental Disorder (SLC6A1-NDD). With permission from the administrators, TREND Community gathered all postings between January 2016 to February 2021 from a private social media group anonymously, and analyzed them for key terms. The number of mentions of concepts related to disease burden were analyzed for a 12 month period (February 2020 to February 2021) of conversations, and all of the conversations. The 20 terms that were most often mentioned are listed, with very little change in frequency between the 12 month period and the longer.

The Community Voice Report also revealed that seizures were the predominant topic of conversation amongst families (Figure 1). The Neurological domain was most frequently discussed, with seizures, epilepsy, absence seizures, tonic-clonic seizures, tremor, atonic, and drop seizures making up 7 of the 20 most often mentioned terms. The other core symptoms of SLC6A1-NDD, including autism and developmental delays were also frequently discussed. Interestingly, patients discussed anxiety, falls, pain, regression, and hyperactivity as often as they discussed developmental delays, demonstrating that these symptoms may have a large daily impact on family life. There was very little change over time in the ranking of concerns discussed, with seizure-related terms mentioned much more often than any other issues.

**FIGURE 2:**
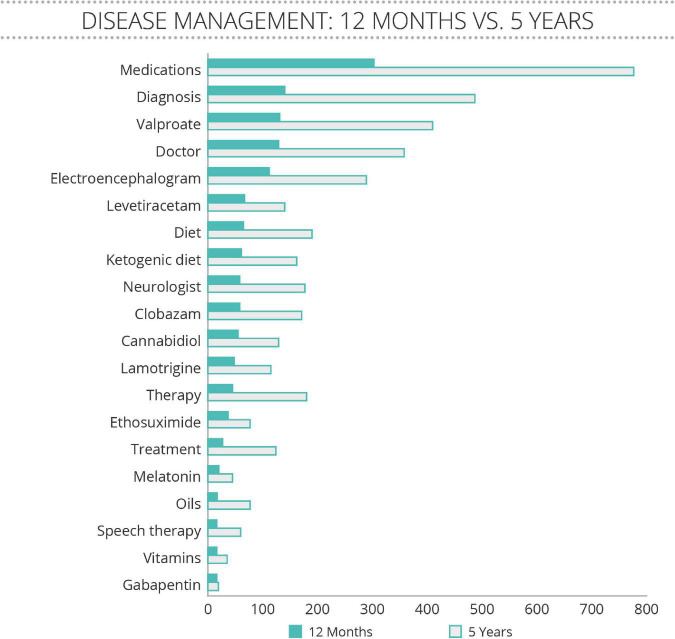
Treatment terms discussed spontaneously by family members of people with SLC6A1 Neurodevelopmental Disorder (SLC6A1-NDD). Analysis for treatment terms of postings on a private Facebook group between January 2016 to February 2021, and for a 12 month period between February 2020 and February 2021. The number of mentions of concepts related to disease management revealed that anti-epileptic medications were discussed more often than any other treatment approach. “Diet” and “ketogenic diet” were ranked in the top 10 and the terms “therapy” and “speech therapy” ranked in the top 20.

Further analysis of these postings revealed that families were often comparing notes on anti-seizure medication regimens (Figure 2). Among the 20 most frequently discussed treatments, anti-seizure medications (including cannabidiol) were the most often mentioned, along with the ketogenic diet, and melatonin. Supplements, vitamins, and other developmental therapies were less frequently discussed.

The Disease Concept Map (Table 1) lists domains and symptoms experienced by people with SLC6A1-NDD without regard to frequency. These additional concepts were included under the Motor and Emotional domains. Additional concepts elicited from the interviews of two KOLs are not included here, as it is not possible to calculate relative frequency from such a small sample.

In addition to the domains of disease for SLC6A1-NDD (Table 1), the impacts of those symptoms on the individual (Table 2) and on caregivers (Table 3) were also cataloged. Proximal impacts included the need for assistance to perform most activities of daily living, as well as risk of falls and injuries. Distal impacts on community and school participation included absenteeism, academic challenges, communication difficulties, safety, and transitioning issues. Socialization and capacity for work was impacted by *SLC6A1* mutation, as well as by the challenging behaviors reported.

Though mutations in the *SLC6A1* gene have significant ramifications on the individual, there are also profound impacts on caregivers, with impacts on work and family life, as well as emotional and financial burdens (Table 3). The time it takes to care for a family member with a neurodevelopmental disorder, such as that caused by *SLC6A1* mutation, interferes with independence, mobility, socialization, completion of household tasks, and the ability of caregivers to work. Feelings of anxiety, stress, depression, and guilt are reported by KOLs and amongst the postings in the social media group. Caregivers report experiencing fatigue caused by the need for constant vigilance, as well as from disrupted sleep. Modifying factors which can reduce the impact of caregiving include access to support services, therapy, and equipment.

## Discussion

The draft conceptual model of SLC6A1-NDD described here, derived from published case reports, analysis of social media interactions, and interviews of two KOLs, demonstrates that the impact of *SLC6A1* variants reaches beyond the hallmark symptoms of epilepsy, autism and intellectual disability. Variants in *SLC6A1* were first linked to a seizure disorder, and epilepsy is still the most prominent symptom as well as the highest concern amongst families. However, as more patients are diagnosed, the spectrum of severity is widening and epilepsy may have less of an impact on daily life experience than the other disease concepts, such as developmental delay and behavioral problems.

Indeed, among the published cases of SLC6A1-NDD, there is a wide spectrum of severity of symptoms. Epilepsy is commonly reported, though the time of onset, severity and type of seizures is variable. Regression has been noted in core abilities, such as motor function and communication, as reported by KOLs, and may be related to uncontrolled epilepsy, but regression has not yet been systematically studied. Other aspects of SLC6A1-NDD beyond epilepsy, such as maladaptive behaviors and communication deficits, may cause more daily burdens on patients and caregivers (Cohen et al., 2022).

The draft conceptual model of SLC6A1-NDD will likely be very similar to that of many other neurodevelopmental disorders, such as Angelman syndrome (Willgoss et al., 2020) and STXBP1-Related Disorder (Sullivan et al., 2022), with only a few distinguishing domains, such as the presence of cognitive regression, and the idiosyncratic hand use described by KOLs as the first noticeable symptom of the disorder. Neither of these domains has been well-studied or reported in the published literature to date. There is growing evidence that rare missense mutations on *SLC6A1* can lead to schizophrenia (Rees et al., 2020) rather than to the neurodevelopmental phenotype described here. In the future, SLC6A1-Related Schizophrenia may well be described as a separate disorder, or added to a spectrum of SLC6A1-related disorders, which could include the other phenotypes such as anxiety and dementia not discussed here.

Currently, there is no accepted treatment for SLC6A1-NDD though many people with SLC6A1-NDD manage their symptoms with dietary modifications, anti-seizure medications, behavioral therapy, physical therapy, occupational therapy, and speech therapy (Devries et al., 2020; Tang et al., 2020). In one study focused on seizure management in children with SLC6A1-NDD, 66% of patients became seizure free while on an anti-seizure medication (Johannesen et al., 2018). Half of these patients took valproic acid, often in conjunction with other seizure medications, and the other half took lamotrigine (Johannesen et al., 2018). It should be noted that current treatments primarily target seizures, and therapeutic interventions addressing the other domains of disease are quite variable.

A recent paper by Bain et al. (2022) compares caregiver-entered phenotypes from 43 patients participating in the Simons Searchlight Registry, with clinician-reported phenotypes from 116 patients reported in the literature, finding that most core features are consistently reported by both groups, with a few exceptions (Bain et al., 2022). Caregivers reported hypotonia as a symptom significantly more frequently than clinicians did, but both groups reported the core domains of autistic traits, developmental delay, and attention deficit hyperactivity disorder (ADHD) at the same rate. Caregivers also reported movement disorders and stereotypies more frequently than clinicians. The authors suggest that there are many missing data points in the provider-reported data because case reports are not standardized. This also illustrates the different perspective of healthcare professionals, whose focus on the medical and neurological aspects of disease may minimize the downstream impacts of the other domains on lived experience of patients and their caregivers.

The Community Voice Report reinforced the importance of the predominant domains of disease identified in the literature (“seizures,” “autism,” “epilepsy,” and “delays”) but the repeated use of terms such as, “anxiety,” “pain,” “tired,” and “worry” suggest the impacts of these symptoms on the family. It is possible that the data collected through social media could be biased by those families who experience the most severe impacts, as parents who have fewer concerns may not be posting as often, nor looking to the patient groups for advice or understanding. It is clear that the symptoms depicted in Table 1 have a significant impact on patient and caregiver quality of life (QoL), however, studies have not yet been conducted on QoL in SLC6A1-NDD. Publications describing impacts on patients and caregivers of people with other similar neurogenetic disorders were used to infer the impacts of these symptoms, as well as the interviews with KOLs (Tables 2, 3). Table 2 outlines the impact of SLC6A1-NDD symptoms on patients’ activities of daily living as well as on socialization and family life. A lack of independence and need for assistance with activities of daily living (ADL) are commonly reported (Freed, 2020). These activities include requiring assistance with bathing, dressing, eating, and toileting. For school-age children with SLC6A1-NDD, KOLs noted problems with absenteeism, academic challenges and the need for adaptive school placements. Additionally, children with SLC6A1-NDD have difficulties with adapting to change in their regular schedule, as well as a reduced sense of danger. There is an impact on family life, as well, with maladaptive behaviors providing a challenge to parent and sibling relationships. Furthermore, people with SLC6A1-NDD have difficulties with social relationships with peers, and as a result, often experience social isolation.

Lastly, interviews with KOLs reveal a social impact on both patients and caregivers, as patients have difficulty engaging with others and caregivers struggle to find the time to maintain their own social relationships (Freed, 2020). Disease concept studies performed for Angelman Syndrome, a disorder typically more severe than SLC6A1-NDD, demonstrate shared characteristics with SLC6A1-NDD such as seizures, speech impairment, ataxia, and intellectual disability (Grieco et al., 2019). Although a final interview-based conceptual model will reveal concepts unique to SLC6A1-NDD, the similarities between these disorders allow for comparison between patient and caregiver impacts. The financial burden of caregiving was reported by KOLs, but economic status was not discussed as a modifying factor, possibly because the emotional impacts of caring for a family member with SLC6A1-NDD overwhelm the issues that can be addressed by increased financial resources. Further description of disease burden on the patient and caregiver are presented in Table 3.

The use of Community Voice Reports to refine the draft conceptual model demonstrates that social media can be a valuable source of insights into the lived experience of rare disease patients and their caregivers. The major limitation of this draft conceptual model of SLC6A1-NDD is that it is drawn only from literature, social analytics, and two KOL interviews, and should be considered as a starting point for a more comprehensive conceptual model of SLC6A1-NDDs which should be based on a large sample of open-ended interviews of caregivers and health care providers.

## Data availability statement

The insight data from analysis of social media interactions, and the raw data supporting the conclusions of this article that were drawn from literature, will be made available by the authors, without undue reservation.

## Ethics statement

Ethical review and approval was not required for the study on human participants in accordance with the local legislation and institutional requirements. Written informed consent from the patients/participants OR patients/participants legal guardian/next of kin was not required to participate in this study in accordance with the national legislation and the institutional requirements.

## Author contributions

TB, KG, AF, MB, and MP conceptualized the study. TB, KH, AP, and MP directed the research. TB, LM, NW, ES, MX, KL, and MP did the research. TB, KL, MX, ES, and MP designed the figures. TB, LM, and NW drafted the manuscript. TB, KG, LM, NW, KH, ES, AP, MX, KL, AF, MB, and MP edited the manuscript. All authors contributed to the article and approved the submitted version.
